# Acclimation lags in alpine grasslands reveal early warning signs of climate change

**DOI:** 10.1111/nph.70638

**Published:** 2025-10-11

**Authors:** Karl Andraczek

**Affiliations:** ^1^ German Centre for Integrative Biodiversity Research (iDiv) Halle‐Jena‐Leipzig Puschstrasse 4 04103 Leipzig Germany; ^2^ Systematic Botany and Functional Biodiversity Leipzig University Leipzig Germany; ^3^ Leibniz Centre for Agricultural Landscape Research (ZALF) Müncheberg Germany

**Keywords:** acclimation lags, adaptation, alpine grasslands, climate change, ecosystem functioning, plant functional strategies

## Abstract

This article is a Commentary on Bektaş *et al*. (2026), **249**: 1173–1187.

Climate is a strong evolutionary driver of ecosystem structure and function – yet, it is changing at an unprecedented pace. This trend is especially pronounced in alpine ecosystems (Pepin *et al*., 2022), triggering a cascade of plant responses. In the short term, plants adjust to novel conditions through phenotypic plasticity, for example by investing in more drought‐resistant tissue. However, in the long term, insufficient adaptation may lead to species turnover. The time lag between these two states – an *acclimation lag* – is crucial for understanding how efficiently communities adjust to novel climates (Bektaş *et al*., 2021). Despite the rapid pace of warming in alpine regions (Myers‐Smith *et al*., 2019), acclimation lags are frequently observed. Yet, the extent to which they are driven by early shifts in plant functional strategies, and how these shifts cascade to ecosystem functioning, remains poorly understood. This is particularly concerning, as changes in ecosystem functioning can feed back onto the plant community, potentially accelerating alpine community change (Bardgett & Wardle, 2010).

*…these findings reveal short‐term acclimation lags in both traits and ecosystem functioning – an early signal of potential maladaptation*.

Plant functional traits help identify ecological trade‐offs above‐ and belowground (Weigelt *et al*., 2021) while providing mechanistic insights into how shifts in plant strategies scale up to ecosystem functions. In an article published in this issue of *New Phytologist*, Bektaş *et al*. (2026; pp. 1173–1187) built on this framework to investigate how short‐term changes in plant strategies mediated early vegetation responses in herbaceous alpine communities to climate change, and how these responses affected ecosystem productivity and litter decomposition. To do so, the authors conducted a 5‐yr transplantation experiment (500‐m downslope) simulating climate warming (corresponding to an increase of 3°C and a 30% longer growing season compared with alpine conditions). In an exceptionally holistic functional approach, they measured key aboveground traits (leaf mass per area, leaf nitrogen and plant height) and belowground traits (root nitrogen, root tissue density, root diameter and specific root length) at the community level, and assessed early vegetation shifts under experimental warming. This allowed the authors to capture key plant strategies above‐ and belowground, such as the fast–slow gradient of resource conservation and the fungal collaboration gradient (outsourcing vs do‐it‐yourself). In doing so, Bektaş *et al*. (2026) show that alpine communities can rapidly shift their functional strategies, yet some essential traits reveal persistent acclimation lags (Fig. 1). If some traits and processes adjust while others do not, what does this mean for the short‐term trajectories of alpine plant communities under climate change?

**Fig. 1:**
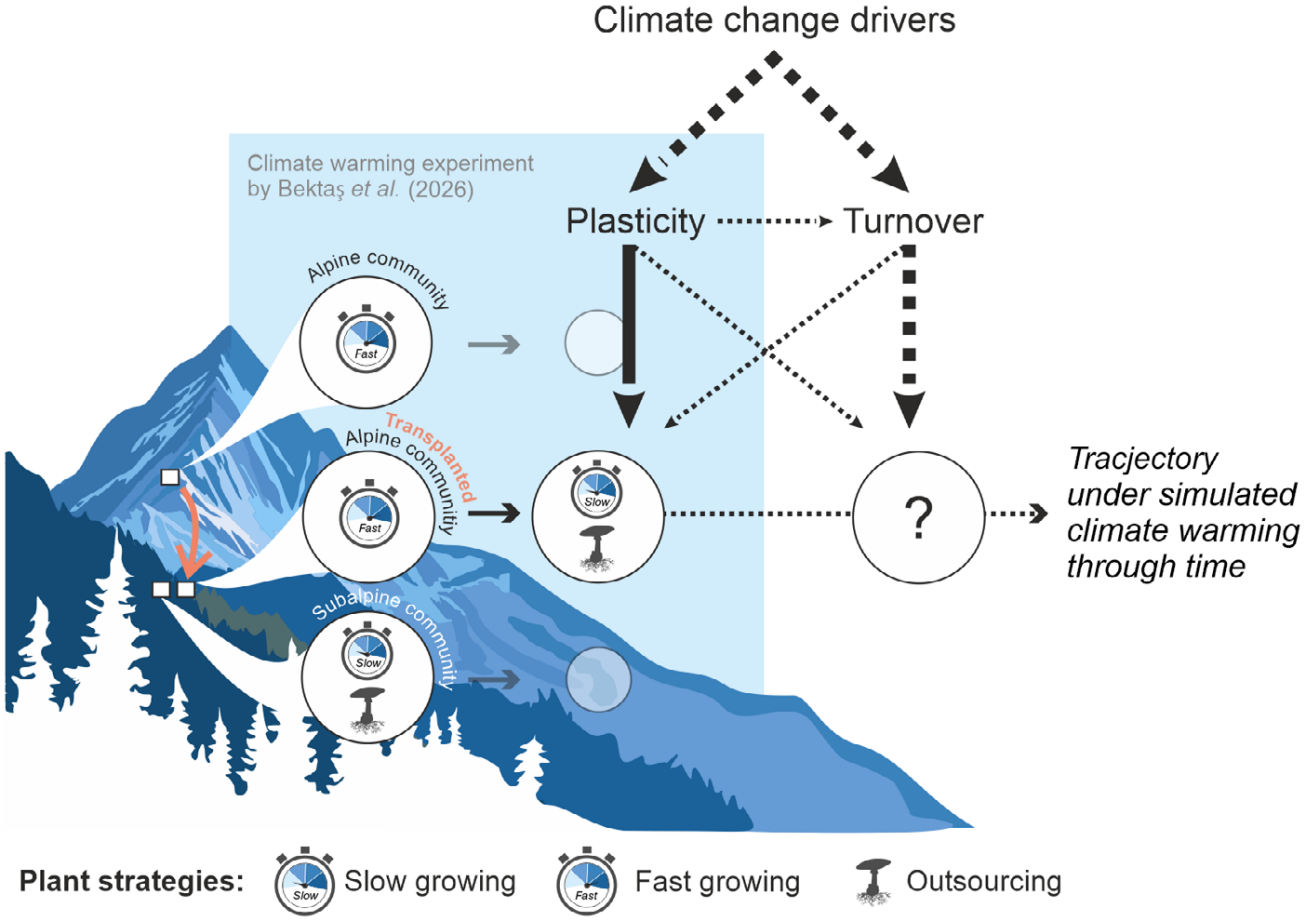
Trajectories of alpine plant communities under climate change. Climate warming can trigger functional shifts within alpine communities (in the short term driven largely by plastic responses), potentially leading to an impairment between traits, ecosystem and microbial functions (Bektaş *et al*., 2026; pp. 1173–1187, in this issue of *New Phytologist*). However, over longer timescales, evolutionary limits to plasticity may lead to species turnover, with uncertain consequences for community composition and ecosystem functioning. The blue box illustrates the simplified outcome of Bektaş *et al*. (2026). Dashed arrows indicate unresolved processes that are critical for predicting long‐term community trajectories. [Correction added on 8 December 2025, after first online publication: some icons used in the figure have been updated.]

## Lifting alpine plant communities into novel climates

Climate warming increases temperature and growing‐season length – two major constraints on the growth and survival of alpine plant communities – thus driving shifts in both community composition and ecosystem functioning (Stemkovski *et al*., 2025). Two contrasting short‐term responses are often expected, largely reflecting changes in species abundance and plasticity. First, in response to climate warming and longer growing seasons, plants may adopt more acquisitive, fast‐growth strategies, typically linked to higher productivity. Alternatively, if warming intensifies water limitation, strategies may shift toward more conservative, stress‐tolerant traits that enhance drought resistance (Myers‐Smith *et al*., 2019). Bektaş *et al*. (2026) show that these trajectories are not mutually exclusive. Warmed alpine communities increased both productivity and litter decomposition, while their strategies shifted from fast to slow and outsourcing. Although this may appear counterintuitive – since slow and outsourcing strategies are usually associated with lower productivity – the authors attribute this to contrasting downslope environmental factors: greater energy input boosted productivity, while increased drought induced plastic shifts toward drought resistance. Thus, warmed alpine communities acclimated to novel climates relative to resident subalpine communities. Interestingly, Bektaş *et al*. (2026) found two key exceptions, which may be pivotal to understanding the short‐term trajectories of alpine communities under climate warming. First, while most traits acclimated to subalpine conditions, one key trait – vegetative height – did not. This suggests that plant species within alpine communities may fail to fully adjust to the novel climates, increasing the risk of species turnover via immigration of taller, more competitive subalpine species. Second, warmed alpine communities showed increased belowground productivity, exceeding that of subalpine communities. Together, these findings reveal short‐term acclimation lags in both traits and ecosystem functioning – an early signal of potential maladaptation.

## The next frontiers in understanding acclimation lags

To better understand these acclimation lags, Bektaş *et al*. (2026) advocate for future research on their key underlying mechanisms – plasticity and species turnover. Plants can adjust their phenotype to novel conditions, but such plasticity has evolutionary limits. If these limits are exceeded, species turnover may become inevitable (Stemkovski *et al*., 2025). Hence, the transition from early vegetation responses due to plastic responses, up to potential tipping points of species turnover, warrants deeper investigation. Inspired by the article by Bektaş *et al*. (2026), I propose key future directions that may lie at the frontier of advancing this goal.

### Distinguishing plasticity from changes in species abundance and turnover

One challenge is to tease apart plastic responses from changes in species abundance and turnover (Fig. 1). To address this challenge, hyperspectral and high‐resolution 3D imagery may help the scientific community to overcome logistical constraints of quantifying aboveground traits for long‐term experiments. However, these methods are less precise in capturing short‐term plastic responses, which may be relatively subtle. An alternative could be phytometer plants (e.g. see De Giorgi *et al*., 2025), which allow the capture of plastic responses of standardized plant species. Species turnover in the warmed alpine communities studied in Bektaş *et al*. (2026) was a minor factor, possibly because of the relatively short duration of the experiment (5 yr). Yet, while one might predict that turnover simply requires more time, another interesting possibility that warrants deeper investigation is whether plasticity has delayed turnover, for example by enhancing the performance of dominant species (Valladares *et al*., 2014; Stemkovski *et al*., 2025).

### Identifying which traits best predict community trajectories

Another key direction is to determine which functional traits best predict the short‐ and long‐term dynamics of alpine communities under climate change (Fig. 1). While plant traits are essential for capturing ecological strategies, selecting the most relevant ones remains challenging (Weigelt *et al*., 2023). Bektaş *et al*. (2026) offer a valuable template for future studies embracing the complexity of whole‐plant strategies, inviting the scientific community to go beyond the traditionally used traits (Matthus *et al*., 2025). For example, long‐term growth and regeneration may depend strongly on clonal traits (Dolezal *et al*., 2020), while rooting depth could be key for capturing impacts of receding permafrost (Blume‐Werry *et al*., 2019). Such traits may also determine the likelihood of turnover as well as vegetation–climate feedbacks – for instance, traits linked to leaf albedo can influence surface temperature and snow cover, potentially buffering climate change impacts.

### Disentangling the multiple drivers of vegetation change

A third challenge is to evaluate the relative contribution of different climate drivers, such as increased growing‐season length vs drought, as *causal factors* to vegetation change (Fig. 1). The experiment by Bektaş *et al*. (2026) is crucial for generating new hypotheses about alpine community trajectories under climate change. Future experimental studies will help us to disentangle these climate change drivers, while long‐term observations offer power to explore additional drivers and their attribution (Dudney *et al*., 2025) on the plasticity and turnover of alpine communities, and potential cascading effects on ecosystem functioning. In both cases, the scientific community should embrace robust statistical models (e.g. fixed‐effects panel regressions from fields outside ecology) to account for confounding factors, such as human impacts, when explaining community trajectories and their effects on ecosystem functioning (Schrodt *et al*., 2025; Siegel & Dee, 2025).

## Conclusion

Bektaş *et al*. (2026) embraced the difficult but essential challenge of elucidating the transient dynamics of alpine plant communities under climate change. Their work sparks numerous interesting questions: how do short‐term acclimatization mechanisms interact with long‐term processes in shaping community trajectories? Can the observed decoupling between traits and ecosystem functioning be generalized across other alpine regions and beyond? Which traits beyond those currently studied are most critical for predicting ecosystem responses under climate change? Joint experimental networks (Bektaş *et al*., 2024), combined with mechanistic modeling of climate drivers (Dudney *et al*., 2025) and their impacts on ecosystem functioning via plasticity and turnover, will be among the frontiers of this research (Myers‐Smith *et al*., 2019).

## Disclaimer

The New Phytologist Foundation remains neutral with regard to jurisdictional claims in maps and in any institutional affiliations.
